# Polyglutamylation: biology and analysis

**DOI:** 10.1007/s00726-022-03146-4

**Published:** 2022-03-31

**Authors:** Cristian I. Ruse, Hang Gyeong Chin, Sriharsa Pradhan

**Affiliations:** 1 Moderna Therapeutics, 200 Technology Square, Cambridge, MA 02139 USA; 2 New England Biolabs, 240 County Rd, Ipswich, MA 01938 USA

**Keywords:** Polyglutamylation, Tubulin, Protein chemistry, NanoESI, Mass spectrometry, Proteomics

## Abstract

Polyglutamylation is a posttranslational modification (PTM) that adds several glutamates on glutamate residues in the form of conjugated peptide chains by a family of enzymes known as polyglutamylases. Polyglutamylation is well documented in microtubules. Polyglutamylated microtubules consist of different α- and β-tubulin subunits with varied number of added glutamate residues. Kinetic control and catalytic rates of tubulin modification by polyglutamylases influence the polyglutamylation pattern of functional microtubules. The recent studies uncovered catalytic mechanisms of the glutamylation enzymes family, particularly tubulin tyrosine ligase-like (TTLL). Variable length polyglutamylation of primary sequence glutamyl residues have been mapped with a multitude of protein chemistry and proteomics approaches. Although polyglutamylation was initially considered a tubulin-specific modification, the recent studies have uncovered a calmodulin-dependent glutamylase, SidJ. Nano-electrospray ionization (ESI) proteomic approaches have identified quantifiable polyglutamylated sites in specific substrates. Indeed, conjugated glutamylated peptides were used in nano-liquid chromatography gradient delivery due to their relative hydrophobicity for their tandem mass spectrometry (MS/MS) characterization. The recent polyglutamylation characterization has revealed three major sites: E445 in α-tubulin, E435 in β-tubulin, and E860 in SdeA. In this review, we have summarized the progress made using proteomic approaches for large-scale detection of polyglutamylated peptides, including biology and analysis.

## Introduction

**Fig. 1:**
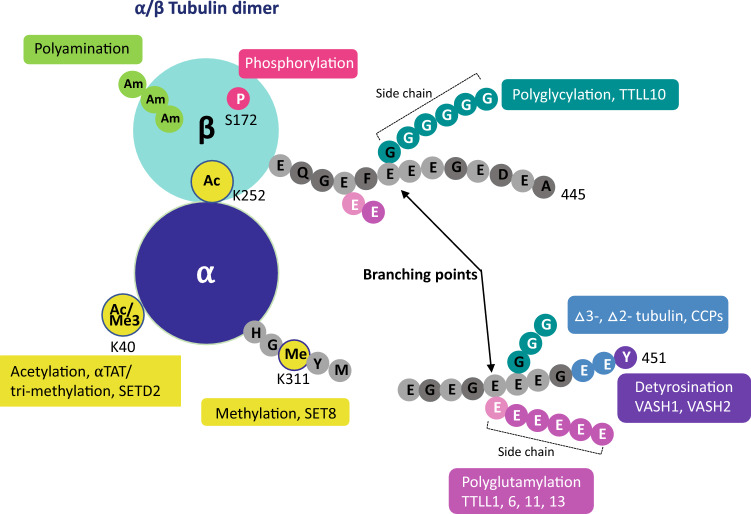
Tubulin heterodimer consisting of α- and β-tubulin with various posttranslational modifications and representative enzymes. Adapted from Magiera and Janke (2014) and modified to include newly discovered PTMs

Polyglutamylation is a posttranslational modification (PTM) that adds glutamates on glutamate residues in the form of conjugated peptide chains by a family of enzymes known as polyglutamylases. These enzymes display both substrate and reaction specificity (Janke et al. 2008; Janke and Kneussel 2010). The glutamylation reaction starts with the formation of a covalent bond between the amino group and the γ-carboxyl group of glutamate in the primary protein chain. The glutamyl side chain then elongates through the addition of successive addition of glutamate residues to the α-carboxyl group of the preceding glutamate (Fig. 1).

Polyglutamylation influences an array of biological functions. The role of microtubule polyglutamylation in neurodegeneration in mouse models and human disease has been recently reviewed (Bodakuntla et al. 2021a). Hyperpolyglutamylation of microtubules, which generates heavily polyglutamylated microtubules, contributes toward neurodegeneration in a mice model. In neurodegeneration diseases, microtubule loss is a common endpoint phenomenon (Bodakuntla et al. 2021b). Proteomic analyses in a neurodegenerative transgenic mouse model revealed an accumulation of insoluble aggregates that contained mostly intermediary filament proteins (Wang et al. 2009). In the insoluble aggregate, a dedicated cytosolic chaperonin, known as CCT, was found to bind actin and tubulin subunits (α1A, α4A, β2A, β5, β4, β2B, β3) (Wang et al. 2009; Horwich et al. 2009). In addition to microtubules, detergent-insoluble aggregates might represent another source of modified tubulin subunits in a proteolysis-resistant milieu (Kopito 2000; Wang et al. 2009; Horwich et al. 2009). Recently, hyperglutamylation of free tubulin was shown to trigger ER stress, and consequently unfolded protein was shown to accumulate in a neurodegenerative mutant (Purkinje cell degeneration, pcd) mouse model (Li et al. 2020). Neuronal nuclear protein induced by axotomy (Nna1) is the major neuronal deglutamylase, and its deficiency leads to hyperglutamylation associated with dysregulation (Li et al. 2020; Bodakuntla et al. 2021b). The proposed catalytic mechanism leading to hyperglutamylation involves glutamylase (tubulin tyrosine ligase-like, TTLL1)/deglutamylase (Nna1) imbalance modulated by site competition of a glycine ligase (TTLL3), and possibly by an undiscovered decyclase (Li et al. 2020). A list of glutamylases, deglutamylases, their protein substrates and bond specificity are presented in Table 1.

**Table 1:** Glutamylases and deglutamylases, protein substrates, and bond specificity

Enzymes	Substrate proteins	Bond specificity
**Glutamylases**		
TTLL1	α-Tubulin β-Tubulin	γ-Linked, initiation
TTLL4	β-Tubulin, preferentially Nucleosome assembly protein 1-like 1 (NAP1L1) Nucleosome assembly protein 1-like 4 (NAP1L4) NF45 protein Protein SET B23 nucleophosmin Acidic nuclear phosphoprotein 32 family, member A (ANP32A) Acidic nuclear phosphoprotein 32 family, member B (ANP32B) Transformation upregulated nuclear protein (RNP-K) Microtubule-associated protein EB1 Ran GTPase activating protein 1 (RANGAP1) 5'-nucleotidase, cytosolic II (NT5C2) BiP protein (GRP78) histone-binding protein (NASP)	γ-Linked, initiation
TTLL5	α-Tubulin, preferentially Nucleosome assembly protein 1-like 1 (NAP1L1) Nucleosome assembly protein 1-like 4 (NAP1L4) NF45 protein Protein SET B23 nucleophosmin Acidic nuclear phosphoprotein 32 family, member A (ANP32A) Acidic nuclear phosphoprotein 32 family, member B (ANP32B)	γ-Linked, initiation
TTLL6	α-Tubulin, preferentially microtubule-associated protein EB1	α-Linked, elongation
TTLL7	β-Tubulin, preferentially Microtubule-associated protein EB1	γ-Linked, initiation α-Linked, elongation
TTLL9	α-Tubulin, preferentially	α-Linked, elongation
TTLL11	α-Tubulin, preferentially	α-Linked, elongation
TTLL13	α-Tubulin, preferentially	α-Linked, elongation
**Deglutamylases**		
CCP1	α-Tubulin, β-tubulin	α-Linked, γ-linked added by TTLL6, only C terminus processing
CCP4	α-Tubulin, β-tubulin	α-Linked, C terminus processing
CCP5	α-Tubulin, β-tubulin	α-Linked, γ-linked, C terminus processing
CCP6	α-Tubulin, β-tubulin	α-Linked, C terminus processing

Furthermore, glutamylation of β-tubulin has been proposed to play a role in neurodegeneration in a different mutant AMS (Ataxia and Male Sterility) mouse (Sheikh et al. 2021). In AMS, Nna1 deglutamylase gene contains a missense mutation in the coding region resulting in substitution of amino acid R808P. In cultured neurospheres derived from Nna1 homozygous mutants β-tubulin displayed hyperglutamylation, which led to increased protein stability in the cerebellum of AMS mice. Indeed, neuronal stem cells from AMS mice showed increased β-tubulin protein levels without changes in β-tubulin mRNA levels. In Nna1 mutation homozygous mouse neurosphere cultures an increased β-tubulin glutamylation and stability was observed in cerebellum of AMS mice. Neuronal stem cells from AMS mice showed increased β-tubulin protein levels without changes in β-tubulin mRNA levels. The authors demonstrated that AMS mutation decreased the NNA1 levels and increased glutamylation in the cerebellum of AMS mice. They hypothesized that the observed changes in glutamylation might alter neuronal stem cell (NSC) properties and the neuron maturation process, leading to Purkinje cell death in AMS mice (Sheikh et al. 2021). Therefore, it is important that modern proteomic assays are applied to detect polymeric modifications, to derive protein sequence, and to quantify peptide levels for both microtubule and detergent insoluble aggregates of tubulins.

Another possible molecular mechanism of hyperglutamylation-induced neurodegeneration suggests that the modification itself impairs the binding affinity of established microtubule interactors and negatively regulates cargo traffic in neurons (Bodakuntla et al. 2021b). Reported the absence of functional deglutamylase (cytosolic carboxypeptidase 1, CCP1) and dysregulated tubulin polyglutamylation were linked to a human neurodegeneration disorder (Shashi et al. 2018). In addition to neuronal disorders, mutations in the tubulin deglutamylase CCP5 have been linked with human vision disorders, while polyglutamylated cellular structures associated with cell division were correlated with cancer (Magiera et al. 2018a). There is currently limited information on the relationship between molecular structures of detected glutamyl side chains and disease phenotype. Site-specific modifications, polymeric unit composition, and the existence of both established and minor substrates (e.g., low [^3^H]glutamate incorporation) need to be characterized and defined at a neuronal level within different brain regions. Since glutamylation and polyglutamylation patterns are a direct result of catalytic activities of both polyglutamylases and deglutamylases, kinetic control and catalytic rates of tubulin modification by these enzymes influence the pattern of functional microtubules (Park and Roll-Mecak 2018). The distribution of cellular polyglutamylated microtubules consist of different α- and β-subunits with defined side-chain length. In other cellular structures, such as axonemes, centrioles, and neurons, the distribution of polyglutamylated microtubules was identified with glutamylation-specific antibodies, GT335 and polyE (Janke et al. 2008; Magiera and Janke 2013).

## Glutamylation enzymes family TTLL and their mechanisms

Molecular modeling and molecular dynamics determined the interaction between C-terminal tail of tubulins (CTT) and glutamylases (Natarajan et al. 2017). Structural studies with site-directed mutagenesis and activity assays compared α-tubulin specific TTLL5 and β-tubulin specific TTLL7 and found highly similar binding modes. The acidic residues anchored the CTT to glutamylases including the site of modification preceded by Gly without binding (Natarajan et al. 2017). It has been suggested that polyglutamylases display selectivity towards glutamate in Gly–Glu motif (Luduena 1993). The current molecular simulations of carboxy terminal tails in tubulins of microtubules include only peptide structures without specific PTM. Mapping the degree of modification is needed for polymeric CTT modifications, polyglutamylation, and polyglycylation using molecular simulations (Schmidt-Cernohorska et al. 2019).

In addition to glutamylase/deglutamylase enzymatic activity, recent studies showed that glutamylation levels can be regulated by the cilia and spindle-associate protein (CSAP). CSAP stimulate both TTLL5 and TTLL7 elongase activity (Bompard et al. 2018). Molecular mechanism studies suggested the involvement of CSAP and TTLL glutamylases in reciprocal regulation of their protein abundances through stabilization. Therefore, the expression of CSAP might provide yet another regulatory mechanism to control glutamylation levels in neuronal microtubules (Bompard et al. 2018). Glutamylation is also enzymatically reversed by the CCP family (Rogowski et al. 2010). The new branch of the polypeptide chain can then be elongated by successive addition of glutamic acid by specific TTLL. In fact, TTLLs are known to specialize either the initiation or the elongation step of the reaction. Biochemical and crystallography studies have shed light on substrate specificity and reaction mechanisms of these enzymes. Although multiple other substrates are also known for polyglutamylation (Table 1), tubulin is the major target of this modification, and its biology is well studied (van Dijk et al. 2008).

Using purified recombinant TTLL4/6 enzyme in biochemical assays with defined tubulin substrates and analyzing the reaction product with tandem mass spectrometry (MS/MS), Roll–Mecak’s laboratory has shown that the glutamylase TTLL4 is specialized in initiating glutamate branches on the β-tubulin tail, while TTLL6 is specialized in elongating glutamate chains on the α-tubulin tail (Mahalingan et al. 2020). In the same study, using nuclear magnetic resonance (NMR), it was demonstrated that the TTLL6-catalyzed glutamate chains are α-linked (Mahalingan et al. 2020). Indeed, purified recombinant mouse TTLL6 has low activity with either α- or β-tubulin tails. The enzymatic activity increased eightfold with a detyrosinated alpha-tail peptide (α1BY), containing a C-terminal glutamate, compared to a C-terminal tyrosine (α1B). A peptide mimicking polyglutamylation on tubulin isolated from neuronal tissue, particularly α-tubulin tails with a monoglutamate branch at either position E443 or E445 displayed 12- to 15-fold higher activity. The activity of polyglutamylation was from the preexisting branch sites, and not from other glutamates of the α-tubulin tail. Therefore, TTLL6 has a strong preference for ligating glutamates to glutamates that have an exposed α-carboxyl group, as opposed to internal glutamates in the tubulin tail that only offers the γ-carboxyl group available for iso-peptide bond formation.

To decipher the TTLL6 polyglutamylation mechanism, crystallography studies were conducted using the conserved catalytic core of mouse TTLL6 (amino acids 51–502), ATP cofactor, and substrate peptides containing analogs for α-elongation, γ-elongation, and glutamylation initiation reaction (Mahalingan et al. 2020). The ATP molecule binds at the interface between the central and C-terminal domains. Structurally, TTLL6 core is similar to glutamylase TTLL7, glycylase TTLL3 and the tubulin tyrosine ligase TTL, demonstrating the structural conservation in the family. For the reaction to occur, the donor glutamate remains nestled in a highly positively charged groove. The conservation patterns of amino acids residue among TTLLs glutamylases suggests a catalytic division between TTLL1, 6, 9,11, 13 as elongases, promoting elongation of the chain, versus TTLL2, 4, 5, and 12 as initiatase (Mahalingan et al. 2020). Indeed, purified recombinant TTLL4 core (amino acids 106–601) activity on unmodified β-tubulin tail peptides adds glutamates to E439 and E440, and does not show the activity on γ-carboxyl added glutamate. These experiments conclusively show a catalytic coordination for polyglutamylation by different class of TTLL enzymes as shown in Table 1.

## Other enzymes that perform polyglutamylation

Proteins with a protein kinase fold are often kinases transferring a phosphate from ATP to substrates generating phosphorylated product and ADP byproduct. In the kingdom of life, phosphorylation is a major PTM affecting protein structure and function. Gram-negative bacteria, *Legionella pneumophila*, meta-effector protein SidJ, successively employ SidE family ADP ribosyltransferase (ART) and phosphodiesterase (PDE) activities to catalyze the covalent ligation of ubiquitin (Ub) to Ser residues on substrate proteins independent of E1 or E2 Ub-activating enzymes (Bhogaraju et al. 2016; Qiu et al. 2016; Kotewicz et al. 2017). SidJ has a putative protein kinase domain spanning amino acid residues 336–593. In an in vitro experiment, phosphorylation of SdeA by SidJ was not observed (Black et al. 2019). In the same series of experiments, co-expression of SidJ, SdeA and calmodulin resulted in a mass increase of 129.9 Da on SdeA. Further, liquid chromatography–tandem mass spectrometry (LC–MS/MS) analysis identified peptides of SdeA modified by one or two glutamates. This glutamylation reaction of SidJ on SdeA was time dependent and preferred Glu to Gln, Asp, Lys, or Gly. In the same report, SidJ also glutamylated SdeB, SdeC, and SidE (Black et al. 2019). To determine the molecular determinants of protein glutamylation catalyzed by SidJ, mutagenesis experiments were conducted in amino acids residues at the canonical kinase catalytic cleft and in the migrated nucleotide-binding pocket, and enzymatic activity on SdeA substrate was measured in vitro. The polyglutamylation activity of SidJ was completely abolished in K367A, D542A, and H492 mutants, and was severely impaired in the N534A mutant, suggesting that the canonical kinase-like active site (K367A or D542A) or at the migrated nucleotide 258 binding site (H492) are involved in the catalytic activity (Sulpizio et al. 2019a).

## Polyglutamylation in tubulin isoforms

Tubulin polyglutamylation is well characterized. The C-terminal region of α- and β-tubulin is flexible and projects outward from the rest of the dimer. Many PTMs occur in the C-terminal region including phosphorylation (β-tubulin), polyamination (α-tubulin), polyglutamylation (α-, β-tubulin), polyglycylation (α-, β-tubulin), detyrosination (α-tubulin), deglutamylation (α-tubulin), acetylation/trimethylation (α-tubulin) and monomethylation (α-tubulin) creating diversity (Fig. 1) (Joe et al. 2009; Park et al. 2016; Chin et al. 2020). These changes result in structural heterogeneity that makes CTT a challenging target for crystallographic studies. Consequently, a recent cryo-electron microscopy study determined the structure of hexameric spastin complexed with a 10-residue peptide of alternating glutamate and tyrosine residues (EY)_5_, as a substitute for tubulin CTT sequences (Han et al. 2020).

## Negatively charged CTT and their roles in regulation of motor traffic

CTT of tubulins interact with microtubule-associated proteins and molecular motors (Hirokawa and Noda 2008). The microtubule surface is covered with a layer of negatively charged tubulin tails specific to each neuronal region. The neuronal organization include the distribution of tubulin PTMs resulting in precise and robust intracellular transport (Park and Roll-Mecak 2018). Motor traffic and its associated defects have been linked to neurodegenerative diseases (Magiera et al. 2018a; Park and Roll-Mecak 2018). For example, kinesin motors were able to distinguish heterogeneity of microtubules (MT) and select PTMs. KIF5-cargo depended on acetylated MT, while kinesin-2 (KIF17) and kinesin-3 (KIF1A) did not select acetylated tracks (Janke and Kneussel 2010). In addition to luminal acetylation (K40), kinesis KIF5 activity showed sensitivity to detyrosination and polyglutamylation of CTT. Elucidating the crosstalk of tubulin PTMs would require isotypes of defined composition in both sequence and modifications. Chimeric tubulins mimicking polyglutamylation showed that kinesin-1 was sensitive to polyglutamylation (Sirajuddin et al. 2014). Two tubulin species were tested: 3 or 10 glutamates crosslinked using maleimide chemistry to a single cysteine in CTTs. This approach of using chimeric tubulins demonstrated that detyrosination regulates both kinesin 1 and kinesin 2 motors (Sirajuddin et al. 2014). Indeed, several studies have demonstrated polyglutamylation regulating the interactions between microtubules and microtubule associated proteins (MAPs), additionally guiding specific motors and cargo (Janke and Kneussel 2010; Gadadhar et al. 2017).

Furthermore, carboxy termini of tubulins are defined by a combination of PTMs including tyrosination/detyrosination, removal of the last two and three amino acids, and polyglutamyl chains. As briefly described by the regulation of motor traffic, polyglutamylation participates in regulatory process in a mixture of tubulin isotypes with diverse modification. The analytical approaches for describing these carboxy-terminal tails share the same amino acid composition with biochemical reactions in a highly negatively charged environment. The primary chain sequence of CTT in α- and β-tubulins accumulate 6 to 12 charges depending on the isoform (Roll-Mecak 2015). Therefore, the baseline negative charge prior to glutamylation reactions is probably larger in CTT primary chain than in many other proteins.

## Deglutamylases and cytoplasmic carboxypeptidases that process glutamates

Both α-linked and γ-linked glutamates from tubulin side chains are processed by tubulin deglutamylases. Tubulin deglutamylases are members of the family of CCP with activities consistent with those of the M14 metallocarboxypeptidase family (Berezniuk et al. 2012; Berezniuk et al. 2013; Kalinina et al. 2007; Wloga et al. 2017). Activity studies suggested that CCPs might require a cofactor (Berezniuk et al. 2013). Indeed, purification of CCPs to homogeneity eliminated its enzymatic activity. Similarly, a bacterial CCP purified to homogeneity for X-ray crystallography studies lacked any enzymatic activity. Among deglutamylases, CCP5 has capability to function as dual-functional enzyme by removing both α- and γ-linked glutamates. Therefore, CCP5 can modulate the effective composition of polyglutamyl chains (elongation), the branching site (initiation) and the truncated tubulins at C-terminus, Δ3 form, removal of the last three amino acids (Berezniuk et al. 2013). Overlapping specificity with CCP5, the CCP1 deglutamylase cleaves longer glutamyl residue structures but does not remove the γ-linked glutamyl at the initiation site. Effectively, deglutamylase CCP1 can participate in a physiological equilibrium to the activity of glutamylases with a role in neuronal homeostasis (Bodakuntla et al. 2021b). Perturbation of this functional relationship led to aberrant activity possibly underlying hyperglutamylation-induced neurodegeneration (Magiera et al. 2018b; Bodakuntla et al. 2021a). Reduced deglutamylase activity resulted in the accumulation of heavily polyglutamylated microtubules, promoting hyperglutamylation that impacts mitochondrial motility (Akhmanova and Hoogenraad 2018; Magiera et al. 2018b; Bodakuntla et al. 2021b).

## Methods for chemical characterization and mass spectrometry

### Brief historical retrospect

In the first decade of discovery of tubulin polyglutamylation and characterization, protein chemistry combined with classical mass spectrometry techniques mapped the modifications in purified microtubules. Initial structural characterization of glutamyl residues of tubulin CTTs involved purification of peptides from limited subtilisin digestion of the tubulin α-/β-heterodimer of pig brain (Redeker et al. 1992). Purified peptides from reverse-phase high-performance liquid chromatography (HPLC) fractions were sequenced by Edman degradation and the ensuing results matched with mass spectra determined by fast atom bombardment mass spectrometry (FAB-MS). FAB-MS required methylation of free carboxylic groups prior to ionization and analysis. These results further confirmed the discovery of glutamylation of CTTs and mapped both tyrosinated and detyrosinated peptides (Edde et al. 1990; Redeker et al. 1992). The presence of tyrosine was confirmed by liquid scintillation counting of [^14^C]tyrosine-labeled tubulin by tubulin tyrosine ligase (Rüdiger et al. 1992).

Early glutamylation assays used ultraviolet absorbance (214 nm) from anion exchange separated synthetic peptides to follow the reaction with *Crithidia* polyglutamylase (Westermann et al. 1999a). When applied to mammalian brain tubulin, 6 M urea SDS-PAGE separation of α- and β-tubulin was required prior to LysC digestion for mass spectrometry analysis. Most abundant species were detected with 15–17 glutamyl residues added to α-tubulin derived peptides. Generation of glutamylated CTT without preparative SDS-PAGE indicated higher concentration of up to 50 glutamyl residues with an average of 35 residues, even though data were not shown for this experiment (Westermann et al. 1999b). Monoisotopic mass-to-charge (*m/z*) values of singly charged molecular ions [M + H]^+^ of α- and β-tubulin CTTs were determined with FAB-MS on a high-field magnet instrument (Rüdiger et al. 1994). Species with 1–7 glutamates were characterized by limited digestion with subtilisin. Isolated pig brain tubulins were shown to contain 1–4 or 1–7 added glutamyl residues for α-tubulin and β-tubulin respectively.

FAB (fast atom bombardment) or liquid secondary ion (LSI ionization) constituted one of the primary methods of protein mass spectrometry before implementation of soft ionization techniques, ESI and matrix-assisted laser desorption ionization (MALDI). FAB-MS analysis introduced polar, charged molecules into the gas phase for mass analysis of biological molecules to approximately 5000 Da (Seifert and Caprioli 1996). Typically, peptide digests were analyzed by introduction from a viscous liquid matrix, such as glycerol, thioglycerol and glycerol–thioglycerol mixtures. Sensitivity of the measurement was dependent on the chemical nature of the analyte and its surface charge. Hydrophilic and hydrophobic interactions depend on surface concentration of the analytes. The intensities of peptide molecular ions [M + H]^+^ in a mixture depend on the hydrophilic index (HI) of the peptide (Seifert and Caprioli 1996). The hydrophobicity/hydrophilicity of peptides determined the ionization efficiencies and detection in ESI as compared to FAB-MS (Hemling et al. 1990). The authors of this early study compared the interfaced LC and chromatographic integrity between FAB-MS on a magnetic sector mass spectrometer and ESI on a quadrupole analyzer. ESI–MS produced better sequence coverage from larger peptides mostly due to multiple charged ions detection that reduced the scanning mass range (Hemling et al. 1990). FAB-MS confirmed phosphorylation in class IIIβ tubulin and identified the Ser444 location (Alexander et al. 1991; Eipper 1974).

FAB-MS analysis of tubulins from limited subtilisin digests identified CTT fragments close to 1.6 kDa while the expected mass was around 4 kDa (Edde et al. 1990; Rüdiger et al. 1992; Alexander et al. 1991; Redeker et al. 1992). MALDI-MS analysis of limited subtilisin digests identified both truncated tubulins from pig brain and a mixture of α- and β-tubulin carboxy-terminal peptides (Rüdiger et al. 1995). Negative ion mode spectra in reflectron mode identified up to 8 glutamyl residues added to β-tubulin CTT peptides. Linear time-of-light (TOF) positive ion mode analysis of truncated α- and β-tubulins independently confirmed the main subtilisin CTT fragments were around 1.6 kDa (Rüdiger et al. 1995). Purified C-terminal peptides for α1/2 and α4 Isotypes expressed in adult rat brain showed specific polyglutamylation of tubulin isotypes (Redeker et al. 1998). Negatively charged peptides were selected by charge interaction with arginine following limited digestion with LysC. Further reversed-phase HPLC purification and MALDI-TOF linear and negative ion mode mapped glutamylation of tyrosinated and detyrosinated peptides between *m/z* 2000 and *m/z* 4000. The missing gap, a very polar derivatized glutamylated amino acid, was not released from filter in the amino acid sequencing by Edman degradation. However, it revealed the location of the modified amino acids. The α4-tubulin showed glutamylation of two sites (E443 and E445) while α1/2-tubulin had only one glutamylation site (E445). Moreover, the α4-tubulin displayed extensive polyglutamylation (11 residues) compared with α1/2-tubulin (Redeker et al. 1998). The MS spectra also showed that more than 75% of α-tubulin was nontyrosinated.

Protein chemistry and MS-based methods mapped extensive polyglutamylation of tubulins in *Trypanosoma brucei* and *Tritrichomonas mobilensis*, respectively (Schneider et al. 1997, 1998). In *T. mobilensis*, α-tubulin did not participate in tyrosination/detyrosination cycle and showed the presence of 4 glutamylation sites with additional 2 sites in β-tubulin (Schneider et al. 1998). Flagellar and stable microtubules in *T. brucei* showed the same glutamylation patterns with iso-peptide bonds at residues E445 (α-tubulin) and E435 (β-tubulin) (Schneider et al. 1997). In *T. Brucei*, the β-tubulin sequence had a carboxyl-terminal tyrosine. In a subsequent study, it was established that the same carboxypeptidase TbVASH detyrosinated both α- and β-tubulin tails of *T.brucei* (van der Laan et al. 2019).

## Structural characterization of polyglutamylation peptide species

In nature, dipeptides, and tripeptides with γ-glutamyl linkage are ubiquitously present. For example, chemical characterization described γ-glutamyl dipeptides in urine (γ-glutamylleucine, γ-glutamylisoleucine, γ-glutamylvaline) and in plants in which glutamic acid bound to several amino acids in protein. Ophthalmic acid is a tripeptide resembling glutathione, l-γ-glutamyl-l-α-aminobutylglycine, abundant in crystalline lens and plant tissue. The structure of ophthalmic acid was discovered by separation on paper electrophoresis. The mobility of γ-linked peptides separated at twice the value for α-linked peptides and the ophthalmic acid displayed the expected γ-linked peptide mobility. Furthermore, the fermentation broth of *Corynebacterium glutamicum* (also known as *Micrococcus glutamicus*) contains three γ-l-glutamyl peptides (Vitali et al. 1965). Both α- and γ-glutamyl dipeptides of l-glutamic acid, l-valine and l-leucine were synthesized and compared with isolates. The paper chromatographic separation was similar for natural and synthetic γ-glutamyl dipeptides and confirmed the linkage of naturally occurring γ-glutamyl dipeptides (Vitali et al. 1965). There are numerous examples of γ-glutamyl peptides described in literature. Modern gas chromatography–mass spectrometry (GC–MS) coupled with specific esterification of the γ-glutamyl allows for linkage determination and quantification of glutathione and ophthalmic acid tripeptides (Bollenbach and Tsikas 2020). This study characterized oxidized glutathione hexapeptide using electron-capture negative-ion chemical ionization GC–MS (Tsikas and Duncan 2014; Bollenbach and Tsikas 2020).

The linkage structure of α-tubulin CTT polyglutamylated peptides was determined by C18 reversed-phase HLPC with synthetic peptides (Redeker et al. 1991). The possible linkages for diglutamylation (γ1α1, γ1γ2) and triglutamylated (γ1α2α3, γ1γ2γ3, γ1α2γ3, γ1γ2α3, γ1γ2α2) tubulin CTT peptides were compared with α-tubulin CTT peptides isolated from mammalian brains. These studies showed the first glutamyl residue to be linked to the γ-carboxyl group of primary chain E445 and the following additional glutamates were amide-linked to the α-carboxyl group of the first glutamate residue (Redeker et al. 1991).

## Advances in glutamic acid/glutamate mass spectrometry and nanoESI proteomics approaches

**Fig. 2:**
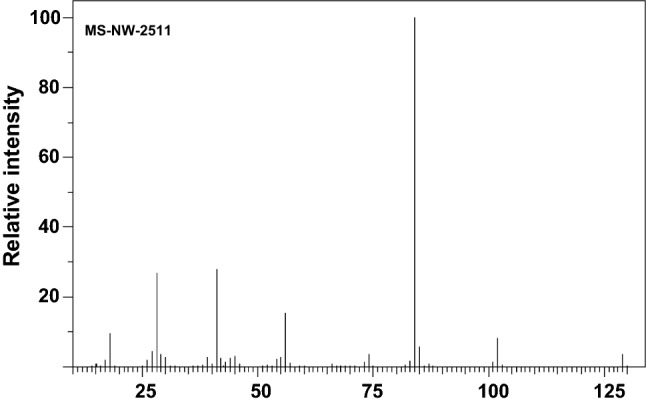
Electron ionization (EI) mass spectrum of nonderivatized L-glutamic acid. The EI mass spectrum was generated on a 75-eV double-focusing mass spectrometer by direct introduction. From: Spectral Database for Organic Compounds SDBS, compound: MS-NW-2511, SDBS NO. 1097. https://sdbs.db.aist.go.jp/sdbs/cgi-bin/landingpage?sdbsno=1097

Figure 2 shows the electron ionization (EI) mass spectrum of native L-glutamic acid. The ion with *m/z* 147 is the radical molecular cation (M^+·^). Additional mass fragments are *m/z* 18.0 (9.5%), *m/z* 28.0 (26.7%), *m/z* 41.0 (27.7%), *m/z* 56.0 (15.3%), *m/z* 84 (100%) and *m/z* 102 (8.1%). The *m/z* 84 ion (C_4_H_6_NO^+^) results from several fragmentation processes and losses of two water molecules and one carbon monoxide molecule (Harrison 2001). The *m/z* 84 ion is most likely pyroglutamic acid immonium ion, but other structures are possible depending on multiple fragmentation mechanisms (Dookeran et al. 1996). The *m/z* 84 ion can be used as a diagnostic mass fragment in (poly)glutamylation studies in CTT peptide MS/MS spectra in modern quadrupole-orbitrap instruments (McClung et al. 2020).

One of the early studies on peptide polyglutamylation with nanoESI used peptide retention time matching between endogenous and synthetic peptides corresponding to tyrosinated (EGEGEEEGEEY), detyrosinated (EGEGEEEGEE) and Δ2(EGEGEEEGE) -tubulin-α1A/1B CTTs (Sahab et al. 2012). These peptide species have different ionization efficiency, and their MS signals cannot be used in quantitative analyses. Neutral loss of water molecules (18 Da) is present in all these peptides and indicated dehydration of γ-carboxylic groups of glutamates. Peptide retention time matching complemented the analysis of fragmentation patterns. These data (i.e., retention time and MS/MS spectra), substantiated the characterization of tyrosinated and detyrosinated tubulin CTT. Retention times of bovine detyrosinated/glutamylated α1A/1B-tubulin were then compared following confirmation with native, nonmodified synthetic peptides. Selection of synthetic peptides started with the separation of tubulin isoforms using immobilized pH gradient (IPG) strips (pH 4–7) in the first dimension, and a 4–12% PAGE in the second dimension. Separated protein species from mouse brain tissues were initially digested with trypsin and then with proteinase K, before sequencing. This study provided identification of E441 and E443 glutamylation sites in mouse and bovine brain α1A/1B-tubulin.

## Analysis of α- and β-tubulins by intact protein mass spectrometry

The functional role of polyglutamylation displays a reversible threshold in response through spastin activity that differs from canonical crosstalk signaling mechanisms. In contrast to single covalent modifications (acetylation, phosphorylation, methylation) with activation/deactivation cycles, it is the number of glutamyl residues per chain that modulates microtubule severing. For biochemical assays, the mean glutamate number was determined from the average intact mass of tubulin species (Valenstein and Roll-Mecak 2016). Three human tubulin (α1B,βI and βIVb) isoforms were monitored for the degree of glutamylation. Calculated numbers of glutamates per chain provided biochemical evidence for spastin assay (mean glutamate number < *n*^E^ > as baseline for any enzyme activity prior to incubation with spastin was calculated as described by Valenstein and Roll-Mecak 2016). The weighted mean of the number of glutamates was calculated from LC–MS of limited subtilisin digestion of α/β-tubulins (Valenstein and Roll-Mecak 2016). The analytical assay for the tubulin by intact mass measurement provided evidence of intact protein molecule successive graded glutamylation through a recombinant enzyme reaction. The newly added or removed glutamates by respective enzymes, namely TTLL7 or spastin, can be measured as the difference from the baseline mean glutamate number prior to addition of these enzyme. The authors used a similar strategy to monitor the role of α-tubulin acetylation defined by the shift of average molecular mass together with the detyrosination shift (Valenstein and Roll-Mecak 2016).

## Mapping polyglutamylation with nanoESI LC–MS/MS

**Fig. 3:**
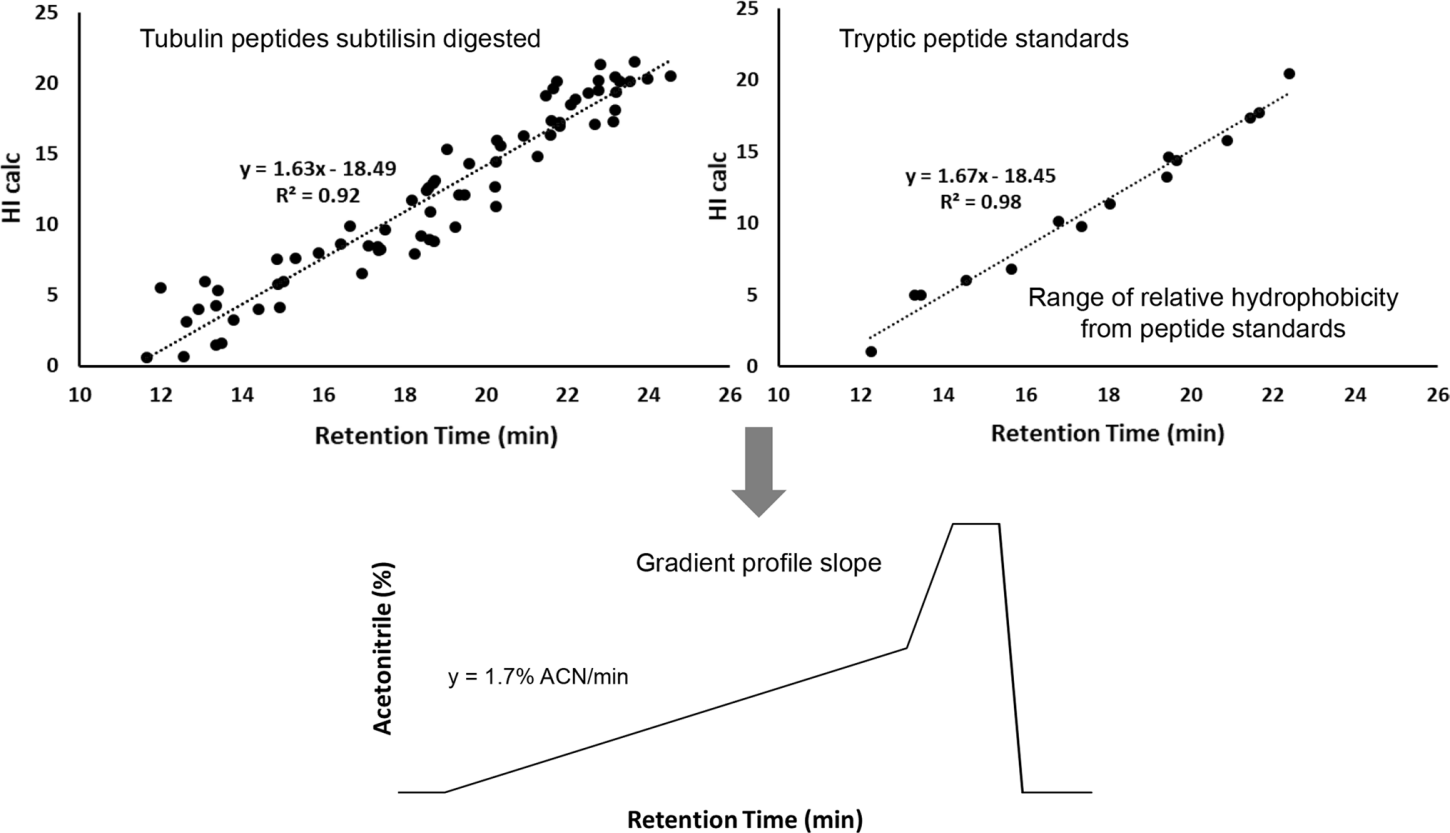
Gradient profile of acetonitrile (ACN/min) in nanoLC–MS/MS (lower panel) of both subtilisin-digested (upper left panel) and trypsin-digested (upper right panel) unmodified tubulin peptides. The graphs illustrate the utility of calculated hydrophobic index (HI calc) as an indicator of the percentage ACN/min gradient in nanoLC–MS/MS. The hydrophobicity of the glutamylated peptides was validated from the reproducibility of the ordered elution of synthetic peptide standards. The line slope for hydrophobic index in relation to retention time of standards reflects the organic solvent gradient programmed for HPLC separation. A similar slope value can be determined from the analysis of tubulin peptides digested by subtilisin. Once established the elution of nonspecific digested peptides mirrored the separation parameters, that would allow one to compare the relative hydrophobicity of peptides with expected glutamyl residue values from the tryptic standard peptides

In our study, we applied a parallel digestion protocol with trypsin and subtilisin to map polyglutamylation of CTT in α- and β-tubulin isolated from porcine brain (McClung et al. 2020). Biochemical digestion was coupled with qualitative nanoLC analysis based on synthetic peptide standards for retention time reproducibility. We obtained extensive sequence coverage from unmodified α- and β-tubulin peptides. The peptide elution profiles of unmodified sequences can be correlated to experimental values. The hydrophobicity index (HI) corresponds to the acetonitrile percentage content required for peptide elution from the reversed phase chromatography column. The HI can provide a unification of peptide hydrophobicity scales and simplify comparison of methods and data between laboratories (Grigoryan et al. 2013; Krokhin 2012). Plotting the calculated HI (HI calc) values versus the retention time of unmodified peptide obtained from subtilisin-digested tubulin resulted in a slope value of 1.63 (Fig. 3). Analogous, synthetic tryptic peptides standards had a slope of 1.67. Considering the hydrophilic nature of the column reversed phase material, both these values approximated the gradient profile slope (1.7% ACN/min) in nanoLC–MS/MS (Fig. 3). Gradient elution profile matched the expected values for both trypsin and subtilisin digests from unmodified α- and β-tubulin peptides. Subsequently, we calculated the relative hydrophobicity of modified conjugated peptides using their added linear sequences. The relative hydrophobicity of polyglutamylated peptides matched the ordered elution of synthetic peptide standards within the expected range (McClung et al. 2020). NanoLC–MS/MS gradient delivery, relative hydrophobicity and predicted retention time are critical parameters for large-scale proteomics analyses for discovery of MS/MS clusters prior to amino acid sequence identification (Beer et al. 2004). In our assay, we proved that calculation of relative hydrophobicity of polyglutamylated tubulin peptides complement structural fragmentation of glutamyl modifications (McClung et al. 2020).

## Proteome assays for discovery of glutamylation in bacterial ubiquitin ligases

*Legionella pneumophilia* delivers approximately 300 effector proteins by its Dot/Icm (defect in organelle trafficking/Intracellular multiplication) secretion system to ensure replication of this infectious pathogen (Hubber and Roy 2010). For intracellular survival, *L. pneumophilia* takes control of membrane rafts, cholesterol-rich microdomains in the host (Manes et al. 2003; Foster et al. 2003). Cholesterol regulates membrane fluidity in eukaryotes and fatty acid composition in bacteria controls membrane fluidity, while both rafts and *L. pneumophilia* membranes contain GPI-linked proteins (Manes et al. 2003; Watarai et al. 2001). Glutamylation of effector proteins SidE on the *Legionella*-containing vacuole surface has been described recently in a series of studies (Sulpizio et al. 2019b). In the next paragraphs, we will discuss the discovery of glutamylation of E860 in SdeA by SidJ/CaM glutamylase.

In a label-free quantitative proteomics assay of immunoprecipitation of GFP-tagged SidJ in HEK293T cells it was found that SidJ requires calmodulin as cofactor (Bhogaraju et al. 2019). SidJ purified from *Legionella* protein quantity was at least twofold higher than any of the ubiquitin ligases (SdeA, SdeB, SdeC, SdeD) using intensity-based absolute quantification, or iBAQ. Immunoprecipitated GFP-SdeA co-expressed with SidJ in HEK293T cells contained the peptide ^855^HGEGTESEFSVYLPEDVALVPVK^877^ from the catalytic region below detection level relative to control. Multiplex TMT quantification showed almost uniform mono-glutamylation of E860 and minor in vitro sites at E857 and E862. Comparison of extracted ion chromatograms showed E860 to be the preferred mono-glutamylation site, although di-glutamylation was also observed. Similarly, low level of mono-glutamylation species was observed at E857/E862 (Bhogaraju et al. 2019).

Flag-tagged SdeA (563–910, mART domain) was purified from HEK293T cells co-expressing GFP or GFP-SidJ (Gan et al. 2019). Silver-stained gel bands were processed and analyzed for digested peptides using LC–MS/MS. Samples purified from GFP-SidJ co-expressed cells showed glutamylation of the peptide ^855^HGEGTESEFSVYLPEDVALVPVK^877^ from the Flag–SdeA–mART domain. The glutamylation of E860 was more than 90% and a low percentage of di-glutamylation was detected. Site occupancy was determined from extracted ion chromatogram areas of modified peptides normalized to an unmodified peptide. Comparison of SdeA and SdeA (Glu860Ala) showed the incorporation of [^14^C]glutamate is site-specific at E860. The same peptide ^855^HGEGTESEFSVYLPEDVALVPVK^877^ was found to contain the major glutamylation signal localized at E860 in SdeA^ΔNC^ (178–1000) isolated from *E. coli* after co-expression with calmodulin and SidJ (Black et al. 2019). Peptide spectral counts quantified up to 6 glutamate residues in Myc-tagged SdeA immuno-isolated from HEK293 cells expressing WT SidJ relative to an inactivated SidJ mutant. The incorporation of additional glutamate residues was restricted to E860 with no signal in E860A mutant detected from [^14^C]glutamate marker (Black et al. 2019).

A different approach for discovering of polyglutamylation of SdeA used cells grown in SILAC (Stable Isotope Labeling by Amino acids in Cell culture) media (Sulpizio et al. 2019a). Heavy isotopically labeled cells co-expressing GFP-SdeA/mCherry-SidJ were compared with GFP-SdeA/mCherry control grown in light SILAC Arg and Lys media. The SdeA catalytic site peptide ^855^HGEGTESEFSVYLPEDVALVPVK^877^ signal was depleted in isotopically labeled SidA/SidJ purified with GFP nanobeads. The proline fragmentation ions specific to the catalytic region peptide mapped to the sequence of the heavy sample and an additional mass of 129.043 Da. Three modified peptides from primary backbone detected mono-, di- and tri-glutamylation of E860. Di-glutamylated E860 of the catalytic SdeA peptide had twice the abundance of mono- and tri-glutamylated species (Sulpizio et al. 2019a). In vitro glutamylation with [^14^C]glutamate of SdeA and SdeA (Glu860Ala) confirmed the MS/MS identification of the unique modification site E860.

**Fig. 4:**
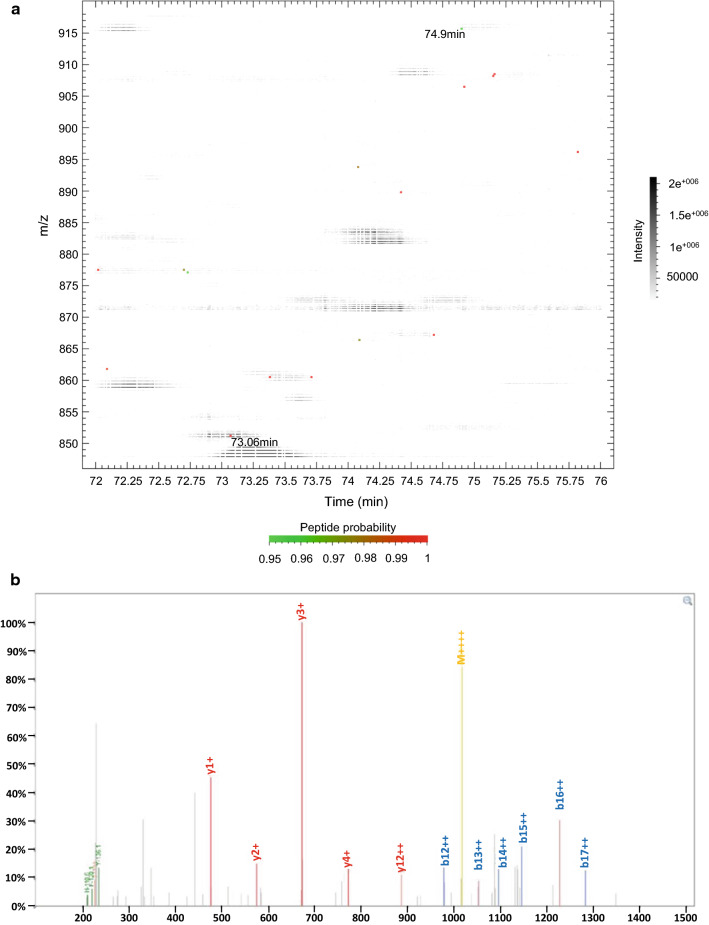
**a** Representation of a di-glutamylated SdeA peptide and its unmodified species from the dataset by Bhogaraju et al. (2019). The data were re-analyzed with TPP/COMET. Color-coded PeptideProphet probability values are represented on the peptide map. Unmodified TMT-labeled peptide QVGRHGEGTESEFSVYLPEDVALVPVK (*m/z* 850.9593, [M + H]^+^ 3400.8153) eluted at 73.06 min and the di-glutamylated TMT-labeled peptide QVGRHGEGTESEFSVYLPEDVALVPVK (*m/z* 915.4806, [M + H]^+^ 3658.9005) at 74.9 min. **b** MS/MS spectrum of di-glutamylated TMT-labeled peptide QVGRHGEGTESEFSVYLPEDVALVPVK (*m/z* 915.4806, [M + H]^+^ 3658.9005) re-analyzed from the dataset published previously (Bhogaraju et al. 2019)

In comparison with tubulin polyglutamylation, there are only a few examples of analytical data from SdeA polyglutamylation species. We re-analyzed the data from a recent study (Bhogaraju et al. 2019) using TPP/COMET. The di-glutamylated QVGRHGEGTESEFSVYLPEDVALVPVK TMT-labeled peptide eluted at 74.9 min, while the unmodified peptide eluted earlier at 73.06 min (Fig. 4). Similar elution times may be characteristic of the di-glutamyl addition. Further studies with unlabeled peptides can enable matching of both calculated and empirical hydrophobicity.

## Protein substrates for polyglutamylation

The elegant study of proteome screening for substrates of TTLL4 and TTLL5 polyglutamylases described a subset of proteins including histone chaperones and regulators of transcription (van Dijk et al. 2008). Nucleophosmin 1 (NPM1), one of the substrates, is part of the NPM protein family that also contains nucleoplasmin 2 (NPM2). High-resolution mass spectrometry revealed that NPM2 is polyglutamylated. Glutamylated peptides were separated by nanoLC-MS/MS at about 60 nL/min flow rate, following pressure loading and offline desalting (Onikubo et al. 2015).

## Conclusions and future perspectives

Polyglutamylation is an important posttranslational modification (PTM) influencing many biological functions. Polyglutamylation and hyperglutamylation in microtubules in mouse models of neurodegeneration have provided useful information. Yet, the significance of polyglutamylation in human disease is still elusive and warrants further investigations. Investigations of polyglutamylation with proteomic approaches has played a considerable role, thus far, and will continue to uncover new protein targets and characterize the modification sites.

Space proximity near interactors of TTLL polyglutamylases might provide a potential list of substrates (Redwine et al. 2017). For sensitive analysis, it is conceivable low nanoflow implementation (sub 100 nL/min) will continue to be useful in the detection of unique peptide glutamyl linkage signatures. Alternatively, unconventional peptide MS technologies could provide new analysis venues for the characterization of γ-glutamyl peptide extensions. This potential venue for analysis will most probably require specialized method development. Polyglutamylated peptide analysis could be complemented with absolute quantification of derivatized amino acids, including glutamic acid and glutamate, using GC–MS (Bollenbach and Tsikas 2020) and GC–MS/MS (Deutsch et al. 1999; Xu and Chance 2007).

Nonetheless, the ability to access unique hydrophobic/hydrophilic ranges of γ-glutamyl peptides could provide supplementary information to established nanoESI techniques. The characterization of polyglutamylated peptides with discovery proteomics assays will provide an updated list of protein substrates of polyglutamylases (Janke and Magiera 2020). Driven by nanoLC-MS/MS separation technologies, the identification of new substrates can be measured together with the hydrophobicity of γ-glutamyl linked peptides. LC–MS/MS proteomics approaches for large-scale detection of amino acid conjugated peptides in polyglutamylation, will need to integrate unbiased discovery and validated physico-chemical features. The current context of polyglutamylation characterization uncovered in detail three major sites: E445 (α-tubulin), E435 (β-tubulin) and E860 (SdeA). Variable-length polyglutamylation chains were mapped with a multitude of protein chemistry and proteomics approaches. Additional information to these sites resulted from minor abundance signals present mostly on the same peptide structures. For all three main substrates α-, β-tubulin, and SdeA, a radioactive ([^14^C]glutamate) marker provided either complementary information or site validation. Probable limits of physico-chemical properties of conjugated glutamyl peptides in mixtures are only beginning to be explored in LC–MS/MS proteomics (Bhogaraju et al. 2019; Black et al. 2019; Gan et al. 2019; Sulpizio et al. 2019a). It is expected that the extension of the analytical advances made over the last years in the numerous PTM, notably including methylation and acetylation, would be useful in the area of polyglutamylation.

## Abbreviations

AMS: Ataxia and male sterility
ART: ADP-ribosyltransferase
CCP1, CCP5: Cytosolic carboxypeptidase 1, cytosolic carboxypeptidase 1
CSAP: Cilia and spindle-associated protein
CCT: Cytoplasmic chaperonin containing TCP-1
CTT: C-terminal tail of tubulins
ESI: Electrospray ionization
MALDI: Matrix assisted laser desorption ionization
FAB-MS: Fast atom bombardment mass spectrometry
GC–MS: Gas chromatography–mass spectrometry
HI: Hydrophobicity index
LysC: Endoproteinase LysC
MS/MS: Tandem mass spectrometry (MS/MS)
Nna: Neuronal nuclear protein induced by axotomy
PTM: Posttranslational modification
SILAC: Stable isotope labeling by amino acids in cell culture
TTLL: Tubulin tyrosine ligase-like
TMT: Tandem mass Tag
Ub: Ubiquitin
